# Identification of stem-like cells and clinical significance of candidate stem cell markers in gastric cancer

**DOI:** 10.18632/oncotarget.6890

**Published:** 2016-01-12

**Authors:** Xiaowei Zhang, Ruixi Hua, Xiaofeng Wang, Mingzhu Huang, Lu Gan, Zhenhua Wu, Jiejun Zhang, Hongqiang Wang, Yufan Cheng, Jin Li, Weijian Guo

**Affiliations:** ^1^ Department of Medical Oncology, Fudan University Shanghai Cancer Center, Shanghai, China; ^2^ Department of Medical Oncology, The First Affiliated Hospital of Sun Yat-Sen University, Guangzhou, Guangdong, China; ^3^ Department of Cancer Chemotherapy Center, Zhoushan Hospital, Zhejiang, China; ^4^ Department of Oncology, Shanghai Medical College, Fudan University, Cancer Hospital of Fudan University, Shanghai, China

**Keywords:** cancer stem cell, gastric cancer, stem cell marker, CD44, CD133

## Abstract

The existence of gastric cancer stem cells (CSCs) has not been definitively proven and specific cell surface markers for identifying gastric CSCs have largely not been identified. Our research aimed to isolate potential gastric CSCs and clarify their clinical significance, while defining markers for GCSC identification and verification. Here, we report that spheroid cells possess stem cell-like properties, and overexpress certain stem cell markers. CD133 or CD44-positive cells also exhibit properties of CSCs. The expression of Oct4, Sox2, Gli1, CD44, CD133, p-AKT, and p-ERK was significantly higher in metastatic lesions compared to that in primary lesions. Elevated expression of some of these proteins was correlated with a more aggressive phenotype and poorer prognosis, including Oct4, Sox2, Gli1, CD44, and p-ERK. Multivariate Cox proportional hazards model analysis showed that only CD44 is an independent factor. Knockdown of CD44 down-regulated the stem cell-like properties, which was accompanied by the down-regulation of p-ERK and Oct4. Oct4 overexpression could reverse the decreased CSCs properties induced by CD44 knockdown. Taken together, our research revealed that spheroid cell culture, and CD133 or CD44-labeled FACS methods can be used to isolate gastric CSCs. Some CSC markers have clinical significance in predicting the prognosis. CD44 is an independent prognostic factor and maintains the properties of CSCs in CD44-p-ERK-Oct4 positive feedback loop.

## INTRODUCTION

Gastric cancer is one of the most common malignancies throughout the world, yet the mechanisms underlying the carcinogenic development of gastric cancers remains poorly understood. The prognosis of the patients with gastric cancer is typically poor, with an average 5 year survival rate of less than 30%. Most of the patients die from metastasis and treatment failure. Therefore, there is an urgent need to improve the understanding of the mechanisms that lead to gastric cancers, so that new treatment strategies may be developed to target these. Recently, researchers have focused on identifying and targeting CSCs. Studying CSCs may improve the overall understanding of carcinogenesis and help to explain the frequency of treatment failure for gastric cancers. CSCs are defined as a unique subpopulation of cells that possess self-renewal and differentiation potential, as well as are generally responsible for tumor initiation, invasion, metastasis, and chemo-resistance. Although there is some controversy surrounding the existence of CSCs and the markers that define them, there is increasing evidence for their existence in several tumor types, while some molecular markers have been identified. Furthermore, the detection of stem cell markers and related proteins have been associated with a poor prognosis for various tumors, including gastric cancer [1–5]; some of these may be driving factors for the formation and maintenance of CSCs, which underline their potential clinical significance [6, 7].

CSCs have been isolated from gastric cancers in a few previous studies, but the existence of gastric CSCs has not been definitely demonstrated. CD44, CD24, and/or CD133 have been suggested to be specific cell surface markers of gastric CSCs, but the data concerning these markers have been largely inconsistent [4, 8–12]. To provide more evidence of the existence of gastric CSCs and clarify their clinical significance, we set to isolate CSCs from gastric cancer and investigate the expression of stem cell markers or related proteins in these CSCs and in clinical tissues samples of gastric cancers. At the same time, we analyzed the correlations between the expression of these proteins and the clinicopathological parameters and patient survival to identify factors that may predict the overall prognosis of gastric cancer.

## RESULTS

### Isolated spheroid cells possess CSC-like cells and overexpress stem cell markers

**Figure 1:**
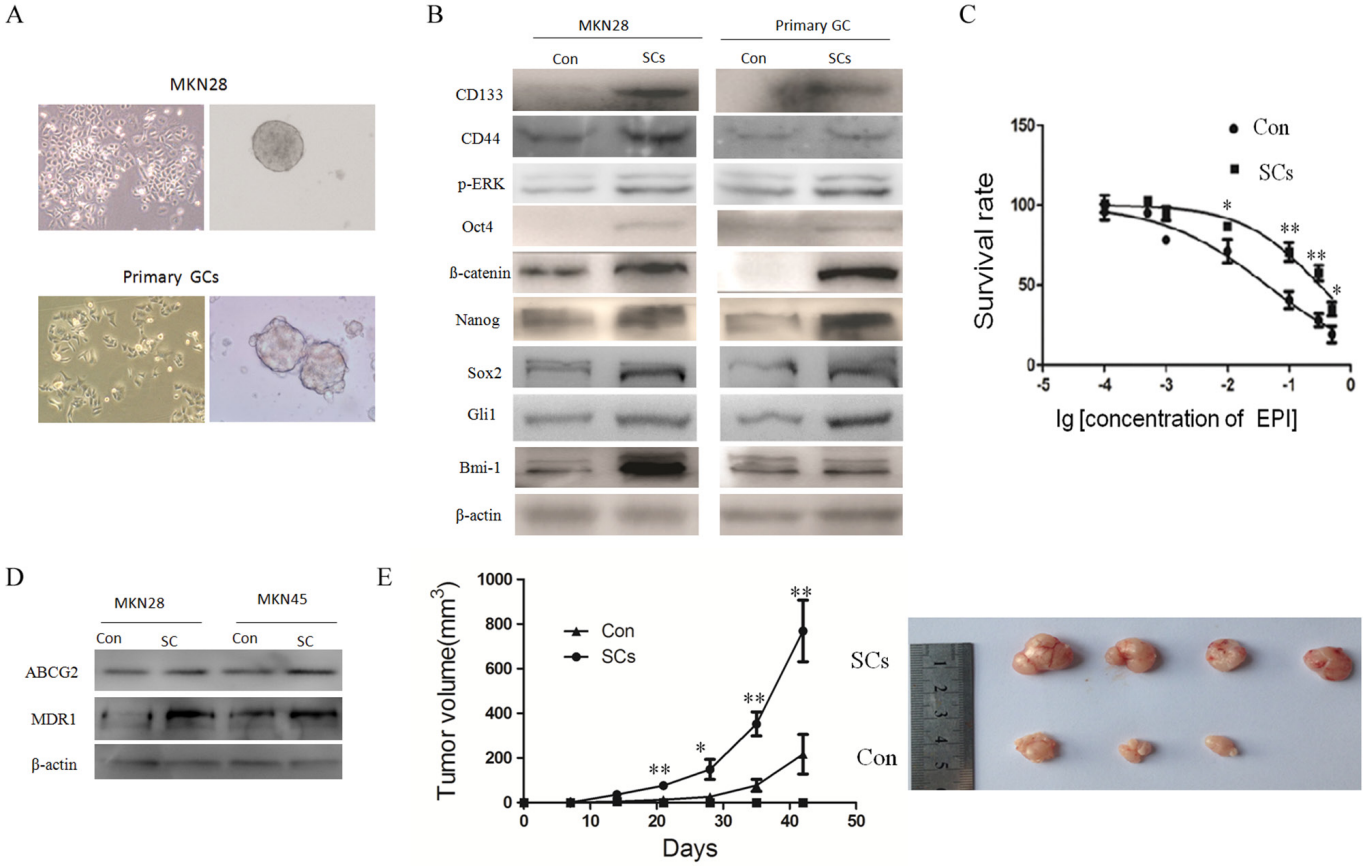
Sphere cells isolated from gastric cancer by serum free culture have the properties of CSCs **A.** tumorigenic spheres are derived from gastric cancer cell lines or primary gastric cancer cells by serum free culture. **B.** isolated spheroid cells overexpress stem cell markers. The expression of stem cell markers in spheroid cells (SCs) from gastric cancer cell lines or primary gastric cancer cells and their parental cells (Con) was detected by Western blotting. **C.** spheroid cells or parental cells are treated with EPI in serum-free RPML-1640 culture medium at the indicated time points (*n* = 5). **D.** isolated spheroid cells overexpress chemo-resistance-related proteins MDR1 and ABCG2, as detected by Western blot. **E.** spheroid cells have higher tumorigenicity *in vivo* compared with parental cells. Pictures of xenografts after subcutaneous injection of control and sphere cells in SCID mice are depicted (*n* = 4). (data are represented as mean +/− SD).

Cultured CSCs are believed to be able to form spheres that have properties, which are very similar to endogenous CSCs isolated from human tumor tissues [13, 14]. Therefore, we cultured GC cells to induce sphere formation, and then we examined the expression of stem cell (SC) markers on these spheres to evaluate the existence of gastric CSCs, while simultaneously identifying new potential CSC markers. We isolated spheres from both gastric cancer cell lines and primary cancer cells isolated from patients, which are often considerably different from established cell lines of similar origin. Four primary gastric cancer cell strains were purified and characterized from four fresh gastric cancer tissues, and three primary gastric cancer cell strains were isolated and characterized from six malignant ascite samples of gastric cancer patients. To do so, four ascite samples were taken after intraperitoneal chemotherapy and two ascite samples were taken before intraperitoneal chemotherapy. Isolated primary GC cells from human tumor tissues or GC cell lines were cultured in a serum-free medium with recombinant epidermal growth factor (EGF) and basic fibroblast growth factor (bFGF). After about 3 weeks, some of the tumor cells grew and formed spheres (Figure 1A). The tumor spheres were maintained in culture for at least 14 days and were passaged 3 times, indicating that the spheroid cells were able to self-renew. Spheroid cells were obtained from the MKN28 and MKN45 GC cell lines. Spheroid cells were also successfully cultured from seven primary GC cell strains isolated from fresh GC tissues or malignant ascites.

Next we examined the expression of previously identified CSC/SC markers in the spheroid cells isolated from primary GC cells. Compared to parental cancer cells, spheroid cells overexpressed CD44, CD133, Oct4, Nanog, β-catenin, SOX2, Gli1 and p-ERK (Figure 1B), which provided evidence for the existence of CSCs and suggested some of these markers might be used to identify gastric CSCs. Similar results were also obtained for spheres cultured from MKN28 GC cell line (Figure 1B). Considering that CSCs are often chemo-resistant, we evaluated the chemosensitivity of these spheroid cells. We ultimately found that spheroid cells were more resistant to chemo-therapy which was accompanied by the overexpression of chemo-resistance related proteins MDR1 and ABCG2 (Figure 1C and 1D). In addition, we tested the tumorigenicity of isolated spheroid cells *in vivo*. The parental and isolated spheroid cells (1 × 10^5^ total cells) were injected subcutaneously into the rear flank of severe combined immunodeficient (SCID) mice and tumor growth was examined. Mice injected with isolated spheroid cells (4 out of 4 mice forms xenografts) formed more and larger tumors within 82 days of injection than those injected with parental cells (3 out of 4 mice forms xenografts) (Figure 1E). This suggested that isolated spheroid cells have higher tumorigenicity, which is considered to be an important and defining attribute of CSCs.

### CD44 or CD133-positive GC cells shows properties of cancer stem cells

**Figure 2:**
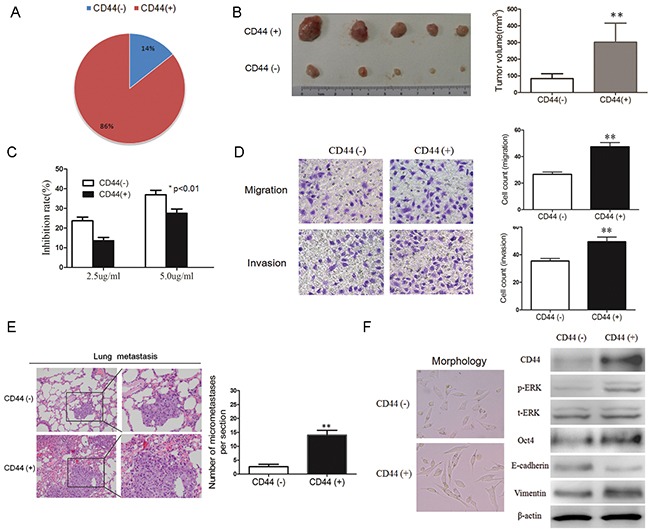
CD44-positive cancer cells have properties of CSCs **A.** CD44-negative (CD44-) and CD44-positive (CD44+) cells were sorted with a FACS Aria (BD Biosciences); CD44-positive cells account for 86% of the total cells. **B.** CD44-positive cells have higher tumorigenicity *in viv*o compared with CD44-negative cells (*n* = 5). **C.** CD44-positive cells display greater chemo-resistance. The CCK8 assay was used to evaluate the sensitivity of CD44-positive or CD44-negative cells to cytotoxic agent 5-Fu treatment. **D.** CD44-positive cells have a higher migration and invasion ability. Transwell migration or invasion assays were analyzed using the Corning chamber. The migration and invasion were photographed (*upper panel*) and quantified (*lower panel*), **E.** Representative H&E-stained sections of the lung tissues isolated from NOD-SCID mice that injected with CD44-negative or CD44-positive cells through the lateral tail vein. The numbers of metastases in the lungs were counted. **F.** Typical EMT morphology photos were shown (left panel); The expression of Oct4, p-ERK, CD44, E-cadherin, Vimentin proteins were detected by western blots analysis in CD44 negative or CD44 positive cells (right panel). (Data are represented as mean +/− SD)

As we have found that two adhesion molecules, CD44 and CD133, were overexpressed in spheroid cells (Figure 1). we aimed to demonstrate that these markers were gastric CSC surface markers. As far as we know, self-renewal and higher tumorigenicity, chemo-resistance, and metastatic ability are hallmark properties of CSCs. Therefore, we evaluated the CSC properties of CD44-positive GC cells from these three aspects. First, CD44-negative and CD44-positive cells in MKN45 cells were sorted with a FACS Aria (BD Biosciences, San Jose, CA), and the percentage of CD44-positive cells was determined to be about 86% in MKN45 cells (Figure 2A), 71.9% in HGC-27 cells, 67.4% in AGS cells and 69.2% in SGC-7901 cells by FACS (data not shown). Then, we performed transplantation into mice to test their self-renewal capacity and found that CD44-positive cells are indeed more tumorigenic than CD44-negative cells (Figure 2B). CSCs has been indicated to be involved in acquiring drug resistance. To examine the chemo-resistance properties of CD44-positive GC cells, we performed cell survival assays by the CCK8 method for MKN-45 GC cells treated with cytotoxic agent 5-fluorouracil (5-FU). Results showed that sorted CD44-positive cells exhibited more chemo-resistance compared to CD44-negative cells (Figure 2C).

We also performed migration and invasion assay using a Matrigel-coated transwell chamber to analyze the metastatic potential of CD44-positive and CD44-negative cells. The results showed that compared to CD44-negative control cells, CD44-positive cells have a higher migration and invasion capacity (Figure 2D). To further explore the role of CD44 on tumor metastasis *in vivo*, CD44-positive and CD44-negative cells were transplanted into NOD-SCID mice through the lateral tail vein. Histologic analysis on the lungs of mice showed that the numbers and size of lung metastasis nodules in CD44-positive group were significantly more and greater than that in CD44-negative group (Figure 2E). These results showed that compared to CD44-negative control cells, CD44-positive cells have a higher metastatic capacity *in vitro* and *in vivo*. Recent evidence suggests that cells that undergo EMT gain stem cell-like properties and, metastatic ability. Here we observed that CD44-positive cells have a more typical EMT morphology (Figure 2F) and lowly express E-cadherin protein and highly express Vimentin protein, which are the two important EMT markers (Figure 2F). In summary, our results revealed that CD44-positive cells appear to undergo EMT and gain stem cell-like properties.

**Figure 3:**
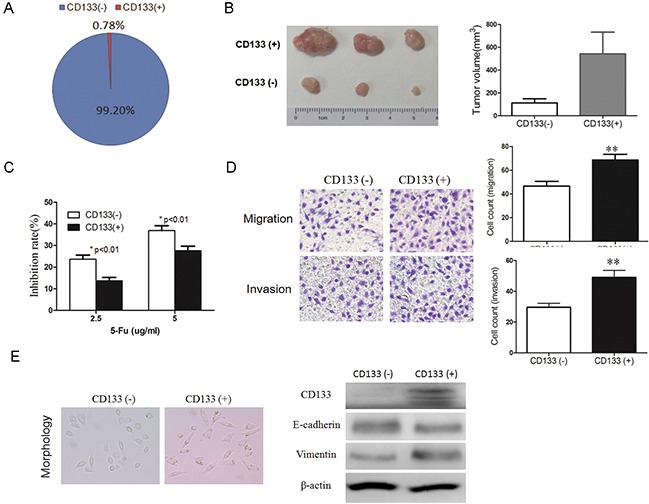
CD133-positive cancer cells have the properties of CSCs **A.** CD133-negative and CD133-positive cells were sorted with a FACS (BD Biosciences) method labeled by CD133 or CD44 antibody, and the percentage of CD133-positive cells in MKN45 cells was 0.78% of the total cells. **B.** CD133-positive cells display higher tumorigenicity *in vivo* compared with CD133-negative cells. Growth curves of tumors after subcutaneous injection of CD133-positive and CD133-negative cells in SCID mice are depicted. Data represent the mean ± SD (*n* = 3). **C.** CD133-positive cells are more chemo-resistance. CCK8 assay was used to evaluate the sensitivity of cytotoxic agent 5-Fu between CD133-positive and CD133-negative groups. **D.** CD133-positive cells has higher migration and invasion ability. Transwell migration or invasion assays were analyzed using the Corning chamber. The migration and invasion were photographed (*upper panel*) and quantified (*lower panel*), **E.** Typical EMT morphology photos were shown (left panel); The expression of CD133, E-cadherin, Vimentin proteins were detected by western blots in CD133 negative or CD133 positive cells (right panel). (Data are represented as mean +/− SD)

We also examined the potential role of the CD133 cell surface marker in identifying and sorting gastric CSC cells. First, we sorted CD133-negative and positive MKN45 cells, and the percentage of CD133-positive cells was about 0.78% (Figure 3A), 1.9% in HGC-27 cells, 2.2% in AGS cells and 2.5% in SGC-7901 cells by FACS (data not shown). Next, we performed transplantation in SCID mice to test their self-renewal capacity, and the results showed that CD133-positive cells were more tumorigenic, compared to CD133-negative cells (Figure 3B). We performed cell survival assays treated with 5-FU, and results revealed that CD133-positive cells exhibited more chemo-resistance compared to CD133-negative cells (Figure 3C). We also performed migration and invasion assays and found that CD133-positive cells has a higher migration or invasion capacity (Figure 3D). We also observed that CD133-positive cells have a more typical EMT morphology (Figure 3E) and lowly express E-cadherin protein and highly express Vimentin proteins (Figure 3E). In summary, our results revealed that CD133-postive cells also possess properties of CSCs.

### Expression difference of candidate stem cell markers in primary lesions and ovarian metastatic lesions of gastric cancer

Immunohistochemical (IHC) analysis was used to detect the expression of candidate stem cell markers Oct4, Sox2, Gli1, CD44, CD133, and potential stemness-related signaling pathway molecules p-AKT and p-ERK in samples from 101 primary lesions of gastric cancer and 72 distant metastatic cancer tissues, of which 21 gastric cancer primary lesions and their paired distant metastasis tissues were included.

**Figure 4:**
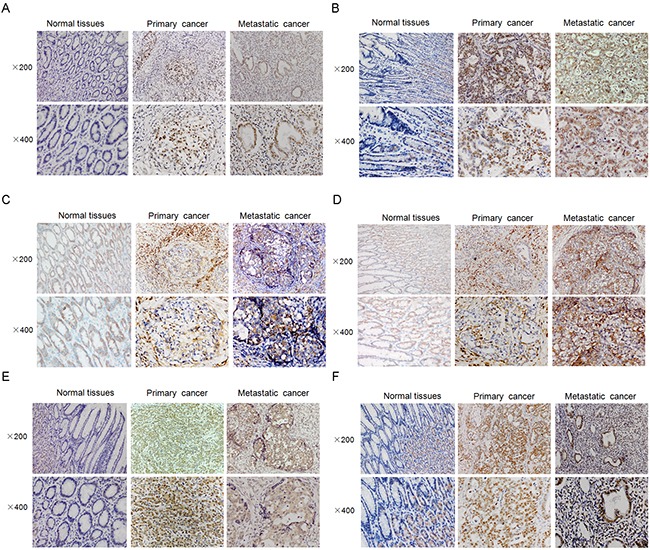
Representative figures of several CSC-related markers or proteins in, gastric tumors, its surrounding normal tissues and paired metastatic cancer samples **A.** primary cancer tissues express less Sox2 compared with the paired metastatic cancer tissues. In panel **B.**, primary cancer tissues express less Gli1 compared with metastatic cancer tissues. **C.** primary cancer tissues expresses less CD44 compared with metastatic cancer tissues. **D.** primary cancer tissues expresses less CD133 compared with metastatic cancer tissues. **E.** primary cancer tissues express less p-AKT compared with metastatic cancer tissues. In panel **F.**, primary cancer tissues expresses less p-ERK compared with metastatic cancer tissues.

We found that expression of Oct4, Sox2, Gli1, CD44, CD133, p-AKT, and p-ERK was significantly higher in metastatic lesions than in primary GC lesions, with a positive rate of original versus metastatic cancer tissues at 59.4% versus 75.0% (Oct4, *p* = 0.003), 52.5% versus 80.6% (Sox2, *p* = 0.000), 53.5% versus 73.6% (Gli1, *p* = 0.007), 42.6% versus 72.2% (CD44, *p* = 0.000), 32.7% versus 70.8% (CD133, *p* = 0.000), 80.2% versus 93.1% (p-AKT, *p* = 0.018), and 35.6% versus 65.3% (p-ERK, *p* = 0.000), respectively (Figure 4).

In the 21 paired specimens, Oct4 and Sox2 expression was significantly higher in metastatic lesions than in primary lesions, with *p* values of 0.016 and 0.031, respectively.

### Candidate stem cell markers expression in primary lesions of gastric cancer correlated with clinicopathologic parameters

The expression of Sox2 was positively correlated with the T (primary tumor) stage (*p* = 0.001) and the sex of the patient (*p* = 0.003). The expression of CD44 was also positively correlated with the TNM stage (*p* = 0.008), vessel invasion, and lymph node metastasis (*p* = 0.043). The expression level of CD133 was positively correlated with TNM stage (*p* = 0.043) and cancer cell differentiation (*p* = 0.024). The expression of Oct4 was positively correlated with lymph node metastasis (*p* = 0.002), cancer cell differentiation (*p* = 0.049), TNM stage (*p* = 0.003), and patient age (*p* = 0.016). The expression of p-AKT correlated with the T stage (p = 0.057) only, while the expression of Gli1 and pERK showed no significant correlations with any clinicopathological factor.

### Prognostic values of each candidate stem cell marker in gastric cancer

**Figure 5:**
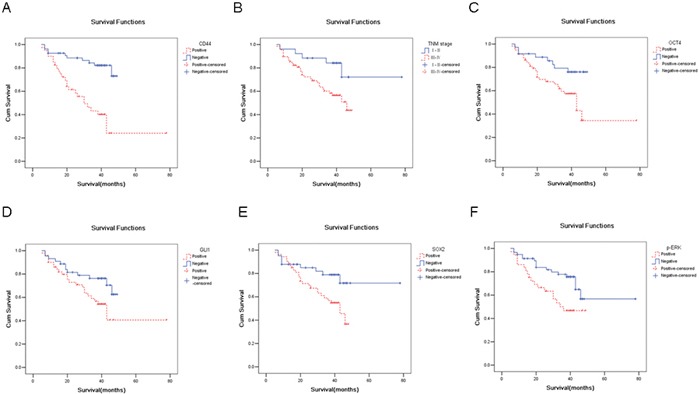
CD44 is an independent prognostic marker In panel **A.**, Kaplan-Meyer survival curves were plotted as cumulative survival versus months according to CD44 expression (negative vs positive). In panel **B.**, Kaplan-Meyer survival curves were plotted as cumulative survival versus months according to clinical stage (stage I/II or stage III/IV). In panel **C.**, Kaplan-Meyer survival curves were plotted as cumulative survival versus months according to Oct4 expression (negative vs positive). In panel **D.**, Kaplan-Meyer survival curves were plotted as cumulative survival versus months according to Gli1 expression (negative vs positive). In panel **E.**, Kaplan-Meyer survival curves were plotted as cumulative survival versus months according to SOX2 expression (negative vs. positive). In panel **F.**, Kaplan-Meyer survival curves were plotted as cumulative survival versus months according to p-ERK expression (negative vs. positive).

The enhanced expression of Oct4, Sox2, Gli1, CD44, and p-ERK predicted a poorer prognosis, with the p-values for each as follows: 0.022, 0.023, 0.045, 0.000, and 0.014, respectively (Table 1, Figure 5). However, there was no correlation between the expression of p-AKT (*p* = 0.3) or CD133 (*p* = 0.124) and survival (Table 1, Figure 5). Multivariate Cox proportional hazards model analysis, which included T classification, lymph node metastasis, distant metastasis, clinical stage, and the expression of Oct4, Sox2, Gli1, CD44, CD133, p-AKT and p-ERK showed that only TNM stage (*p* = 0.011) and CD44 expression (*p* = 0.011) were independent prognostic factors.

**Table 1:** Prognostic implications of candidate stem cell markers expression in gastric cancer

	Total (N)	N of Death	Median survival (months)*	Mean survival (months)	*P* value
**Oct-4**					
Positive	58	26	43	45.1	0.022
Negative	36	8	*	42.4	
**Sox2**					
Positive	53	25	43	35.1	0.023
Negative	41	9	*	62.5	
**Gli1**					
Positive	50	22	43	47.1	0.045
Negative	44	12	*	40.9	
**CD44**					
Positive	40	24	30	37.7	0.000
Negative	54	10		43.5	
**CD133**					
Positive	31	14	43	33.7	0.124
Negative	63	20	*	57.3	
**p-AKT**					
Positive	78	30	*	53.3	0.300
Negative	16	4	46	41.7	
**p-ERK**					
Positive	36	18	34	33.4	0.014
Negative	58	16	*	57.5	

### The correlations between the CSC markers or related proteins

To clarify the correlation between each gastric CSC-related marker and to elucidate the possible regulatory relationships, we analyzed the correlations between each factor and determined the ordinary positive correlation between candidate stem cell markers (Table 2).

**Table 2:** The correlation analyses between candidate stem cell markers

		Sox2	Gli1	CD44	CD133	*p*-AKT	*p*-ERK	
Oct4								
	R	-	0.174	0.213	0.181	0.113	0.051	0.007
	*P* value	-	0.022*	0.005*	0.017*	0.137	0.504	0.922
Sox2								
	R	0.174	-	0.331	0.365	0.292	0.07	0.356
	*P* value	.022*	-	.000*	.000*	.000*	0.36	.000*
Gli1								
	R	0.213	0.331	-	0.245	0.192	0.049	0.325
	*P* value	.005*	.000*	-	0.001*	0.012*	0.518	.000*
CD44								
	R	0.181	0.365	0.245	-	0.415	0.09	0.266
	*P* value	.017*	.000*	0.001*	-	.000*	0.238	.000*
CD133								
	R	0.113	0.292	0.192	0.415	-	0.136	0.201
	*P* value	0.137	.000*	0.012*	.000*	-	0.074	0.008*
p-AKT								
	R	0.051	0.07	0.049	0.09	0.136	-	0.23
	*P* value	0.504	0.36	0.518	0.238	0.074	-	0.002*
p-ERK								
	R	0.007	0.356	0.325	0.266	0.201	0.23	-
	*P* value	0.922	.000*	.000*	.000*	0.008*	.002*	-

The expression of Oct4 was positively correlated with Sox2, Gli1, and CD44 expression. There were also positive correlations between Sox2 and Oct4, Gli1, CD44, CD133, and p-ERK and between Gli1 and Oct4, Sox2, CD44, CD133, and p-ERK. CD44 expression was positively correlated with Oct4, Sox2, Gli1, CD133, and p-ERK, while CD133 expression was positively correlated with Sox2, Gli1, CD44, and p-ERK.

### Knockdown of CD44 negatively regulates the properties of CSCs, accompanied by down-regulation of p-ERK and Oct4 expression

**Figure 6:**
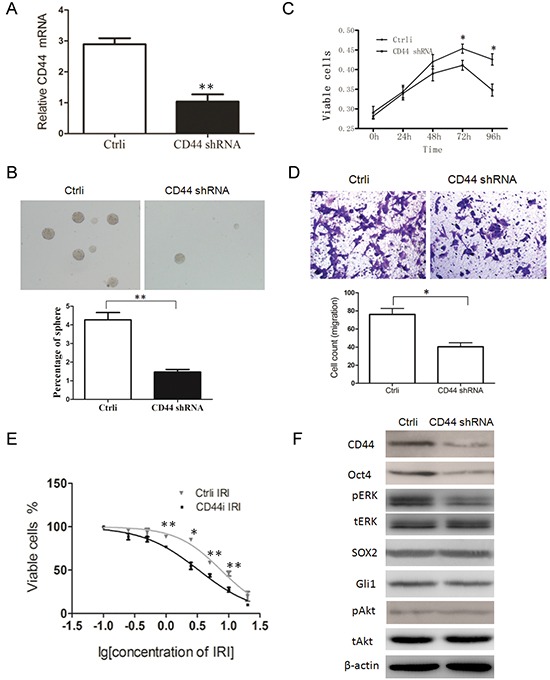
CD44 knockdown negatively regulates the properties of CSCs and the expression of p-ERK and Oct4 **A.** the expression of CD44 mRNA in MKN45 cells transfected with CD44 shRNA or scramble shRNA (Ctrli) was detected by qRT-PCR. **B.** spheroid colony formation ability was reduced by CD44knockdown in MKN28 GC cells. After nearly 3 weeks of culture, CD44 knockdown cells produced significantly fewer spheroid colonies than control cells (transfected with scramble shRNA). **C.** CD44 knockdown cells displayed elevated chemo-sensitivity. The CCK8 assay was used to evaluate the sensitivity of cytotoxic agent 5-Fu between CD44 knockdown cells and control cells. **D.** CD44 knockdown cells have reduced migration ability. Cell migration ability was analyzed using the Corning Transwell chamber. The cells were photographed (upper panel) and quantified (lower panel). **E.** CD44 knockdown cells are more chemo-sensitive. The CCK8 assay was used to evaluate the sensitivity to cytotoxic agent irinotecan (IRI) between CD44 knockdown cells and control cells. **F.** CD44 knockdown downregulates the expression of p-ERK and Oct4. The expression of CD44, Oct4, phosphorylated ERK1/2(pERK), SOX2, Gli1, phosphorylated AKT(pAKT), total AKT(AKT), and actin in control and CD44-knockdown cells was detected by Western blot. (data are represented as mean +/− SD)

As the expression of CD44 is an independent prognostic factor and correlates with every stem cells marker/related protein tested in our study, we suspected that CD44 might maintain the properties of CSCs. To evaluate this hypothesis, we knocked down CD44 expression by Lentivirus-mediated shRNA against CD44 and then examined the properties of CSCs in CD44 knockdown cells compared to those in control cells (infected with control shRNA vector). The knockdown of CD44 in MKN28 GC cells was confirmed by qRT-PCR (Figure 6A), and we determined that CD44-knockdown cells generated significantly fewer spheroid colonies (Figure 6B), exhibited an impaired migration ability, and was more chemo-sensitive than control cells (Figure 6C and 6D), suggesting CD44 maintains the properties of CSCs.

Furthermore, we analyzed the possible regulatory relationship between CD44 and other CSC-related markers, and found that knockdown of CD44 down-regulated the expression of Oct4 and p-ERK (Figure 6F), suggesting that Oct4 and p-ERK are downstream proteins of CD44 and might mediate the functions of CD44.

### CD44 maintains the properties of CSCs via Oct4

**Figure 7:**
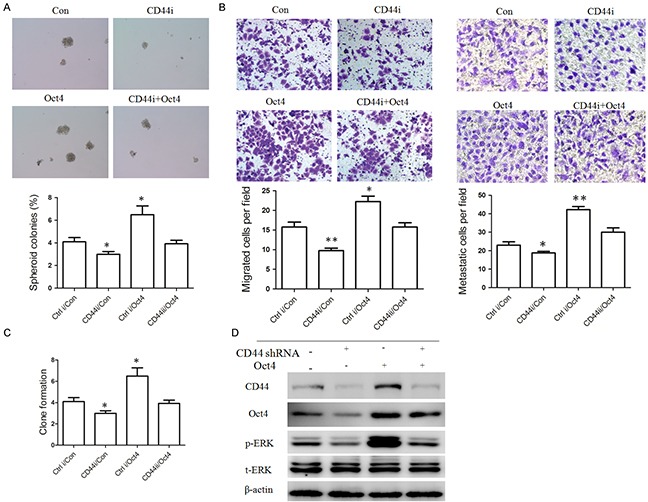
Oct4 is one of the downstream regulator of CD44 **A.** Oct4 overexpression increased the spheroid colonies formation decreased by CD44 knockdown. The spheroid colonies were photographed (upper panel) and quantified (lower panel). **B.** Oct4 overexpression increased cell migration (left panel) and invasion ability (right panel) decreased by CD44 knockdown. The migrated or metastatic cellswere photographed (× 400) and quantified. **C.** Oct4 overexpression partially restored colony formation ability inhibited by CD44 knockdown. The number of colonies were counted and plotted (n=3) (right panel). **D.** The expression of Oct4, CD44 and p-ERK protein in MKN45 cells overexpressing Oct4, CD44 shRNA or Oct4 together with CD44 shRNA was detected by western blots analysis, respectively. (data are represented as mean +/− SD)

Next, we tested whether Oct4 mediate the function of CD44. We co-transfected CD44 shRNA and Oct4 overexpression plasmid and measured sphere-forming ability, migration potential and colony formation of vector-infected control, CD44 shRNA, Oct4-overexpressing, and co-transfected CD44 shRNA with Oct4-overexpressing MKN45 cells by using floating microsphere formation assay, Transwell chamber migration and invasion assays, and plate colony assay. Our results showed that Oct4 overexpression could reverse the decreased transformed phenotypes and stemness (spheroid colonies formation, migration, invasion and colony formation, Figure 7) induced by CD44 knockdown. These data suggest that Oct4 is one of the important downstream mediators of CD44 and CD44 regulates stemness partial via Oct4.

### CD44 upregulates Oct4 through ERK pathway in a positive feedback Loop

**Figure 8:**
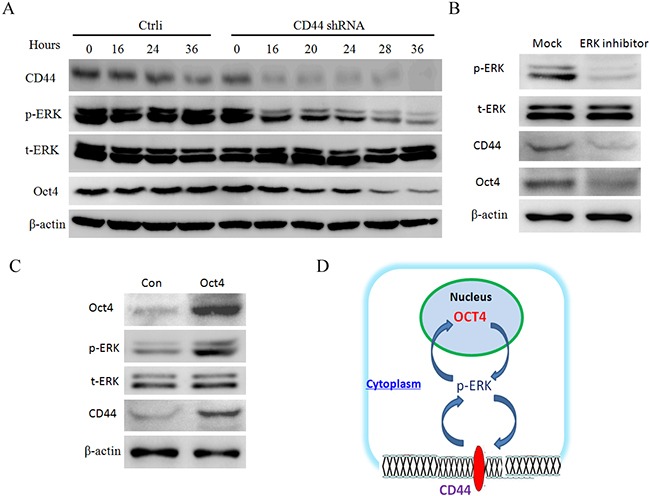
CD44 increases Oct4 expression throug h ERK pathway in a positive feedback loop **A.** CD44 knockddown downregulates p-ERK faster than Oct4 detected by Western blots. over-expression decreases the half-life of Mdm2 protein. Proteins of control and CD44 knockdown SGC-7901 cells were collected at the indicated amounts of time and analysed for the expression of CD44, Oct4 and p-ERK, normalizing to the β-actin. **B.** ERK inhibitor can downregulate both Oct4 and CD44 detected by Western blots. Proteins of control and CD44 knockdown SGC-7901 cells were collected and analysed for the expression of CD44, Oct4 and p-ERK, normalizing to the β-actin. **C.** Oct4 overexpression can upregulate CD44 and p-ERK detected by Western blots. Proteins of control and Oct4 overexpression cells were collected and analysed for the expression of CD44, Oct4 and p-ERK, normalizing to the β-actin. **D.** A diagram of CD44-p-ERK-Oct4 positive feedback loop in maintaining the properties of CSCs. p-ERK acts as an important mediator coupling CD44 and Oct4 to enhance stemness.

Our results showed that CD44 knowdown can downregulate Oct4 and p-ERK (Figure 6F), and CD44 maintains stemness partially via Oct4 (Figure 7). In order to clarify the regulatory relation among three molecules, here we collected the proteins of the CD44 knockdown cells at the indicated time and analyzed change of p-ERK and Oct4 proteins simultaneously. Results revealed that p-ERK protein decreases earlier than Oct4 protein, suggesting p-ERK might be the upstream regulator of Oct4 (Figure 8A).

Furtherly, we next tested whether Oct4 can affect the expression of p-ERK or CD44, and whether ERK inhibitor can affect the expression of Oct4 or CD44. Results showed that ERK inhibitor can downregulates Oct4 and CD44 (Figure 8B), and Oct4 overexpression can upregulates CD44 and p-ERK (Figure 8C). These results revealed that ERK acts as an important mediator between Oct4 and CD44, suggesting an positive feedback between CD44 and Oct4 (Figure 8D).

## DISCUSSION

Better understanding of the relevance and function of cancer stem cells (CSCs) may provide a new understanding and possible targets for GC therapies. CSCs have been previously defined as a small subpopulation of cells that can give rise to tumor masses [15]. CSCs can be viewed as the result of mis-differentiation, and these cells also possess self-renewal and differentiation potential [16]. Recent studies demonstrated that CSCs may be responsible for tumor initiation, invasion, distant metastasis, and chemo-resistance, so the development of therapies that target CSCs are becoming increasingly appealing [16]. Currently, CSCs have been found in many types of solid tumors, such as breast cancers [17], glioblastomas [18, 19], and colon cancers [20, 21], yet there is limited researches that focuses on gastric CSCs. One contributing factor is that some discrepancies exist concerning which proteins may be essential gastric CSCs markers [3, 9, 12, 22–25], particularly in the gastric CSCs isolated from primary GC tissues. Therefore, more research is necessary to support or refute the theory that gastric CSCs are influential to gastric cancer development and treatment resistance; the identification of common biomarkers for gastric CSCs is essential to isolating and studying such gastric CSCs.

For these reasons, we set out to isolate CSCs from GC cell lines and primary GC tissues to pinpoint and characterize available biomarkers for identifying and isolating gastric CSCs via flow. CSCs are believed to be able to form spheres in culture, which then exhibit extensive similarities to endogenous CSCs in human tumor tissues [15]. In this study, we found that spheroid cells from GC cell lines or primary GC tissues possess the ability to self-renewal, and may also play roles in tumor initiation, chemo-resistance, and migration potential. These cells tended to express/overexpress markers of stem cells, such as CD44 and CD133. Therefore, we determined that these cells possess all the hallmarks of CSCs. In this way we have confirmed the existence of gastric CSCs, and we have developed a protocol by which gastric CSCs can be isolated via a serum-free cell culture method. One interesting observation was that two patients from whom we successfully isolated CSC-like cells out of their ascite samples had both received intraperitoneal chemotherapy before isolation. This is consistent with the prevailing theory that anticancer agents can enrich for cancer stem cell-like cells [26]. After determining that these spheroid cells overexpressed CD44 and CD133, we used these markers to identify gastric CSCs via cell sorting and ultimately determined that both CD44-positive and CD133-positive cells possess properties consistent with those of CSCs. Thus, we concluded that these may be useful markers for identifying and isolating gastric CSCs. CD44 and CD133 have regarded as CSCs markers and used as sorting markers for series tumors, including gastrointestinal tumors. CD44 and CD133 have numerous functions, such as supporting cell migration and transmitting proliferation signals, and they are both adhesion molecules that are expressed on the surface of cytomembrane. In a previous study, CD44 was used to identify GC-initiating cells from a panel of human GC cell lines, and CD44-positive GC cells exhibited stem cell properties of self-renewal, as well as the ability to form differentiated progeny to give rise to CD44-negative cells [12]. CD133 has also been used to isolate and purify a subset of CD133-positive cells from GC cell lines that possess at least some properties of CSCs [27]. However, Rocco et al. reported that CD133 and CD44 cell surface markers could not readily identify cancer stem cells in primary human gastric tumors [10].

In our research, the percentage of CD44-positive cells in the MKN45 cell line was 84%, which is fairly high and contradicts the theory that CSCs account for only a small proportion of the total cancer cells, The former research also showed that percentage of CD44 positive cells account for more than 90% in gastric cancer cells detected by FACS [12]. despite the fact that CD44-positive often present with other CSC-related properties. Therefore, we concluded that CD44 can be used as a potential biomarker for gastric CSCs. However, its prevalent expression in cancer cells would prohibit it from being used alone as a sorting marker to isolate gastric CSCs. On the other hand, CD133-positive cells account for only 0.78% of the total population MKN45 cells, which is in agreement with the hypothesis that primary and metastatic tumor masses are generated and maintained by a small subset of tumor cells that capable of self-renewal and thus produce the bulk of the tumor cell mass.

One main weakness of the data supporting the CSC hypothesis is that the markers used to purify CSCs are often not specific enough and therefore in most cases, the CSCs have not been completely purified. Therefore, a more refined selection criteria, such as using a combination of two or multiple markers, is absolutely necessary to improve the purification of CSCs. Although Rocco et al failed to isolate CSCs from human GC tissues by using CD133 or CD44 alone [10], a recent study successfully isolated CSCs from tumor tissues and peripheral blood samples from patients with GC by using a combination of CD44 and CD54 as sorting markers [23]. Thus, our goal going forward would be optimize a combination of cell surface biomarkers to better purify gastric CSCs.

The importance of CSCs in cancer initiation, progression, metastasis, and treatment failure may have clinical significance beyond CSC identification, as the relative expression of key properties could to be used to predict patient prognosis. It has already been determined that the heightened expression of some CSCs markers, such as CD44 and CD133 can be correlated with chemo-resistance, recurrence, and poor prognosis [3, 28].

As we found spheroid cells overexpress multiple stem cell markers, we elected to examine the expression of these candidate CSC-related markers, including Oct4, Sox2, Gli1, CD44, CD133, p-AKT, and p-ERK in GC tissues to assess their clinical significance. Oct4 and Sox2 are both key transcription factors required for maintaining the pluripotency of stem cells [29], and these play crucial roles in the self-renewal of stem-like cells, as well as carcinogenesis and the development of some cancer types. The Gli1 transcription factor is one of the SONIC Hedgehog (SHH) pathway target genes, and it was found that HH-Gli signaling was essential for the maintenance of cancer stem-like cells in human gastric cancers [30] and gliomas [31]. The PI3K/AKT and ERK-MAPK signaling pathways have been investigated extensively and were also found to be essential for maintaining the pluripotency of stem cells, which also promote cancer progress through numerous signaling pathways, including those that regulate the cell cycle and/or inhibit cell apoptosis [32, 33]. pAKT and pERK are activated molecules of these two pathways, and could serve as potential targets for some CSC-trained molecules.

Our results revealed that the expression of CSC-related proteins was obviously correlated with the clinicopathological factors, including lymph node metastasis, tumor staging, pathological type, vessel invasion, nerve invasion, and overall patient survival. The expression of Oct4, Sox2, Gli1, CD44, CD133, p-AKT, and p-ERK was positively correlated with a more aggressive tumor phenotype and poorer overall prognosis. More importantly, the expression of Oct4, Sox2, Gli1, CD44, CD133, p-AKT, and p-ERK was significantly higher in metastatic cancer tissues than in primary cancer tissues. These results suggest that CSC markers and related proteins may play important roles in cancer progression and metastasis, thereby providing clinical evidence to support the existence of CSCs in gastric cancer and the theory that they in turn may help to drive metastasis [34]. Furthermore, some CSC markers may serve as prognostic indicators of survival. In addition, we found that there were positive correlations between these candidate stem cell markers. This phenomenon may due to co-expression of these proteins during CSC development and their coordination/cooperation in the regulation of cancer stem cell development. This observation is evidence of the complex regulation among CSC markers, which points towards intertwining mechanisms that are involved in stemness regulation. p-AKT has been reported to be a downstream target of Oct4 [35, 36], Gli1 [37], and CD4 [38]. However, in our GC tissues, we were unable to detect a significant relationship between p-AKT expression and the expression of other CSC makers. Previous studies have reported that p-ERK can phosphorylate transcription factors, in turn impacting regulation of the cell cycle [39]. In our present work, we found a significant positive correlation between p-ERK expression and Gli1, CD44, CD133, and Sox2; this suggests that p-ERK may be the downstream target of these genes. Furthermore, the expression of p-ERK and p-AKT, which have been reported to effect tumorigenesis in tandem [40, 41] were both highly expressed together in gastric CSCs isolated in this study. There were also positive correlations between CD44 and CD133, also between CD44/CD133 and other CSC-related markers. Several former researches showed that the Wnt [42, 43], PI3K/AKT [44–46], and SHH/Gli1[47] pathways may be activated in CD44 positive and/or CD133 positive cancer cells. Therefore, we hypothesized that CD44 and CD133 participate in maintaining/regulating the expression of other CSC-related proteins. Our results demonstrate the strong positive correlations between CD44 or CD133 and the other detected stem-related markers/proteins.

As so many proteins are overexpressed in stem cells or stem-like cells and can be correlated with metastasis and survival, how can we determine which protein(s) are most important? Although univariate prognostic analysis showed that higher Oct4, Sox2, Gli1, CD44, or p-ERK expression was correlated with a worse prognosis, only CD44 and TNM stage were confirmed to be independent prognostic factors by Multivariate Cox proportional hazard analysis. It suggested that CD44 might be the most important one among these proteins in determining prognosis and predicting survival. Thus, it may serve as a predictive marker for overall survival with a power similar to TNM stage (same *p* value in multivariate analysis in our study) and as a potential therapeutic target for developing new treatment modalities.

Furthermore, CD44 knockdown decreased the stemness of GCs, and the expression of CD44 correlated with all the other stem cell markers tested in GC tissues. CD44 can also positively regulate the expression of Oct4 and p-ERK, both Oct4 [48–52] and ERK1/2 pathway [53] are vital for regulating pluripotency of cancer stem cells. Collectively, CD44 could be a gatekeeper of the cancer stem cells and might be a core regulator of stemness, which can modulate the expression of some important stem cell markers and is co-expressed with others. One recent research has also revealed that CD44 can positively regulate the phosphorylation status of ERK [48, 54]. At the same time, previous studies have also shown that inhibition of ERK decreases CD44 expression and cancer malignancy [55]. We also found the mutual regulatory relationship between CD44 and ERK which suggested that a potential positive feedback regulatory loop exists between CD44 and ERK.

Importantly, it is a novel finding in our study that CD44 is an upstream positive regulator of Oct4, which is an regulater of stemness and promotes cancer cells proliferation and metastasis. Furthermore, Oct4 overexpression could reverse the decreased malignant phenotypes and stemness induced by knockdown of CD44, suggesting CD44 regulates stemness partially via upregulation of Oct4. Intriguingly, our research revealed that Oct4 can also positively regulate CD44 expression, suggesting there might be a positive feedback between CD44 and Oct4. As we known, ERK protein is located in the nucleus and cytoplasm, Transcription factor Oct4 is located in nucleus, while CD44 antigen is a cell-surface glycoprotein. Considering the location of three molecules, we surmised that p-ERK might act as an important mediator coupling CD44 and Oct4. Indeed, we found that p-ERK protein decreases earlier than Oct4 protein in CD44 knowdown cells, and p-ERK could positively regulate Oct4, suggesting p-ERK might be the mediator in the positive feedback between CD44 and Oct4.

Taken together, it is apparent that CD44 maintains the stemness of gastric cancers, and it is not only a cell-surface marker, but also might be a driving factor in the development of CSCs. The more precise mechanisms by which CD44 maintains the stemness of gastric cancers and how the CD44-p-ERK-Oct4 positive feedback loop functions warrants further studies.

## MATERIALS AND METHODS

### Cell culture

Five human gastric cancer cell lines (MKN28, MKN45, NCI-N87, SGC-7901 and AGS) were obtained from the Surgical Institution of Ruijin Hospital. These cell lines were cultured in RPMI-1640 (GIBCO) or DMEM (GIBCO) supplemented with 10% fetal bovine serum (FBS) (GIBCO) and antibiotics.

### Spheroid colony formation assay

Spheroid colony formation assays were carried out as described previously [12]. Human gastric cancer cells were inoculated in each well (10 cells per well or otherwise indicated) of an ultra-low-attachment 96-well plate (Corning Life Sciences, Acton, MA,) and supplemented with 100–200 μl of RPMI-1640 medium (Invitrogen Corp., Carlsbad, CA) plus 10 mM HEPES, 20 ng/ml human recombinant epidermal growth factor (EGF; Invitrogen), and 10 ng/ml human recombinant basic fibroblast growth factor (bFGF) (Invitrogen). After about 3 weeks, each well was examined using a light microscope (BX51, Olympus), and the total well numbers containing spheroid colonies were counted.

### Western blotting

Cells were lysed in AM1 lysis buffer (Active Motif, Carlsbad, CA) and protein concentrations were measured with the BCA protein assay kit (Thermo Fisher Scientific Inc., Carlsbad, CA). Total protein (50 μg) was resolved by 125 g/L SDS-PAGE and transferred onto PVDF membranes. After being blocked in TBST (20 mmol/L Tris, 137 mmol/L NaCl, 1 g/L Tween20, pH 7.6) with 50 ml/L skim milk for 2 h at room temperature, membranes were incubated with CD44, CD133 (Miltenyi Biotech, San Diego, CA), Oct4 (Cell Signaling Technology, Danvers, MA), Nanog (Santa Cruz Biotechnology, Santa Cruz, CA), β-catenin (Cell Signaling Technology), and SOX2 (Cell Signaling Technology), and β-actin primary antibodies (diluted 1:500; Santa Cruz Biotechnology) for 2 h. Membranes were then washed three times with TBST solution, followed by incubation for 1 h with HRP-linked secondary antibodies (1:1000; Santa Cruz Biotechnology) at room temperature. Finally, membranes were visualized using the DAB reagent (Dako Corporation, Carpinteria, CA).

### The establishment of primary gastric cancer cells from fresh gastric cancer tissues or fresh ascite of gastric cancer

Tumor tissues were obtained from patients who underwent gastrectomy for gastric tumors at the Department of Gastrointestinal Surgery and ascites samples were collected from patients with or without intraperitoneal chemotherapy at the Department of Oncology, Shanghai cancer center, Fudan University. Informed consent was obtained from all patients who provided samples, and this study was approved by the Institutional Medical Ethics Committee of Fudan University Shanghai Cancer Center.

### Flow-cytometry analysis and sorting

Cells from different cell lines were incubated on ice for 30 min with the following antibodies: anti-CD44 (1:200; BD Biosciences, San Jose, CA) or CD113 (1:100; Miltenyi Biotec), while isotype was used as a control. The cells were then washed with PBS and centrifuged for 5 min at 1000 rpm. Samples were analyzed and sorted with a FACS Aria (BD Biosciences).

### Transplantation of cancer cells

Various unseparated or purified cell populations were injected subcutaneously into the flanks of SCID mice, as indicated previously [12]. After 30 days, mice were sacrificed by cervical dislocation, tumors were removed, fixed in 10% neutral buffered formalin solution (Sigma), and paraffin embedded. The animal experiment was approved by Institutional Animal Ethics Committee of Fudan University.

### *In vivo* metastasis assays

For *in vivo* metastasis assays, MKN45 cells labelled by CD44 antibody were sorted with a FACS. CD44-positive cells and CD44-negative cells were transplanted into NOD/SCID mice (5-week-old, 4 per group, 1*10^5^ cells for each mouse) through the lateral tail vein. After 8 weeks, mice were sacrificed. Their lungs were removed and subjected to hematoxylin and eosin (H&E) staining. All research involving animal complied with protocols approved by the Shanghai Medical Experimental Animal Care Commission.

### CD44 Knockdown and Oct4 overexpression *in vitro*

The CD44 shRNA constructs in GV248 vector that specifically target human CD44 sequences were purchased from GeneChem Inc. (Shanghai, China). The human CD44 shRNA sequence is 5′-GCCCTATTAGTGATTTCCAAA-3′ [12]. Where relevant, cells were transfected with a control shRNA that does not match any known human or mouse cDNA. The efficiency of infection of the cells was evaluated by a green fluorescent protein (GFP)-expressing plasmid (GV248). Twenty-four hours after transfection, the cells were treated with medium containing puromycin (1 μg/ml; Sigma-Aldrich, St. Louis, MO) to remove non-infected cells. Stable CD44 knockdown clones were pooled and used for further experiments. The vector containing human cDNAs of Oct4 gene was purchased from GeneChem Inc. (Shanghai, China).

### Chemo-resistance experiment

Spheroid cells or FACS-sorted cells were inoculated into 96-well plates (2000 cells per well) in triplicate on the day prior to testing. Each well was supplied with RPMI-1640 medium containing 10% FBS, along with a chemotherapy reagent, such as 2.5 μg/ml or 5 μg/ml 5-fluorouracil (5-FU) (Sigma-Aldrich), 0.25 μg/ml epirubicin (EPI) (Sigma-Aldrich), or 0.5 μg/ml Iritecan (IRI) (Sigma-Aldrich). A no drug control was also included. The appropriate medium for each well was changed 24 h after initial treatment. The number of viable cells was evaluated after 4 days from the initial treatment using the Cell Counting Kit-8 (Dojindo, Rockville, MD) following the manufacturer's instructions, and the optical absorbance at a wavelength of 450 nm was measured for the supernatant of each well using a Multiskan EX plate reader (Thermo Fisher Scientific Inc.).

### Cell migration and invasion assay

Cells were plated in 6-well plates and grown to confluence. RPMI-1640 culture medium (GIBCO) with 10% FBS(GIBCO) and mitomycin (2 μg/ml) was then added to inhibit cell proliferation. Cell migration and invasion ability was analyzed by the Transwell chamber assay [56]. 20% FBS was used as a chemo-attractant. Cells on the lower surface of the insert were fixed and stained and then counted under a light microscope (BX51, Olympus).

### Patients and specimens

101 tissue samples from primary lesions were collected from patients with gastric cancer who underwent surgical operation in Fudan University Shanghai Cancer Center from January 2005 to August 2011. 72 tissue samples of ovarian metastases of gastric origin from April 2004 to August 2011 were collected from the same center. Among them, 21 pairs of tissues were from the same patient. The patients were followed up until November 2011. Written informed consent for the use of tissue samples was obtained from all patients, and the study was approved by the Institutional Medical Ethics Committee of Fudan University Shanghai Cancer Center.

### Immunohistochemical assay

Immunohistochemical (IHC) analyses was used to detect the expression of candidate stem cell markers Oct4, Sox2, Gli1, CD44, CD133, and potential stemness-related signaling pathway molecules p-AKT and p-ERK. Expression of these markers was assessed in samples from gastric cancer primary lesions and distant metastatic lesions. All slides were interpreted by two independent observers in a blinded fashion. If more than 10% of the cells stained moderately or strongly, the cells were considered positive. Otherwise, the sample was considered negative.

### Statistical analysis

All statistical analyses were performed using the SPSS version 16.0 software package (SPSS Inc., Chicago, Illinois, USA). Experiments *in vitro* presented in the figures are representative of three different repetitions. The data are presented as the mean values ± standard derivation. Comparisons between groups were evaluated by Student's t-test. The protein expression difference in gastric primary sites and ovarian metastatic lesions were analyzed by the χ2 test. McNemar tests were used to compare protein expression in the matched primary and metastatic cases. The χ2 test was also employed to analyze the relationships between the protein expression and clinicopathological factors. Survival curves were estimated using the Kaplan–Meier method, and the differences in survival distributions were evaluated by the log-rank test. The Cox proportional hazards model was used to identify which factors may have a significant influence on survival. The relationship between different proteins that were co-expressed was investigated by Spearman analysis. Values of *p* < 0.05 were considered to be statistically significant as indicated.
